# Electrically evoked mismatch negativity responses to loudness and pitch cues in cochlear implant users

**DOI:** 10.1038/s41598-023-29422-1

**Published:** 2023-02-10

**Authors:** Luise Wagner, Anna S. Ladek, Stefan K. Plontke, Torsten Rahne

**Affiliations:** Department of Otorhinolaryngology, Head and Neck Surgery, University Medicine Halle (Saale), University Hospital Halle (Saale), Ernst-Grube-Straße 40, 06120 Halle (Saale), Germany

**Keywords:** Cognitive neuroscience, Perception, Biomedical engineering

## Abstract

Objective measurements could improve cochlear implant (CI) fitting, especially for CI users who have difficulty assessing their hearing impressions. In this study, we investigated the electrically evoked mismatch negativity (eMMN) brain potential as a mainly preattentive response to pitch and loudness changes. In an electrophysiological exploratory study with 21 CI users, pitch and loudness cues were presented in controlled oddball paradigms that directly electrically stimulated the CI via software. Out of them 17 valid data sets were analyzed. A pitch cue was produced by changing the stimulating CI electrodes (pairs of adjacent electrodes). A loudness cue originated from changing the stimulation amplitude on one CI electrode. MMN responses were measured unsing clinical electroencephalography recording according to a standard recording protocol. At the group level, significant eMMN responses were elicited for loudness cues and for pitch cues at basal electrode pairs but not at apical electrode pairs. The effect of deviance direction was not significant and no stimulus artifacts were observed. Recording an electrically evoked MMN in response to loudness changes in CI users is generally feasible, and is, therefore, promising to support CI fitting procedures in the future. Detection of pitch cues would require a greater electrode distance between selected electrodes for standard and deviant stimuli, especially in apical regions. A routine clinical setup can be used to measure eMMN.

## Introduction

A cochlear implant (CI) is a neuroprosthetic device that partially restores the sense of hearing in people with sensorineural hearing loss. It does so by bypassing the cochlea and directly stimulating the auditory nerve via amplitude-modulated pulse trains. During the first few months following activation of the CI, recipients describe auditory percept as artificial^1^ and speech recognition needs to be trained^2,3^. Feedback on individual hearing performance is an important prerequisite for optimal fitting of the CI stimulation. However, for CI users with limited experience or those who are unable to provide responses due to age or other medical conditions, subjective feedback is often limited or nonexistent. The fitting of the audio processor can be supported by objective methods such as electrically evoked stapedius reflex threshold (eSRT)^4,5^ or electrically evoked auditory brain stem responses (eABR)^6–8^, which provide information about auditory thresholds or loudness processing at the brainstem level.

Objective assessment of auditory perception by electrophysiological methods may also provide better insight into the speech perception of the CI users in these cases^9^. These methods are commonly used with normal-hearing subjects^3^. For example, hearing thresholds of normal-hearing and CI users can be objectively estimated using electroencephalography (EEG)^10^. Recording cortical auditory evoked potentials (CAEP) using EEG is particularly beneficial for CI users to avoid interference from artifacts caused by electrical stimulation^11^. Mismatch Negativity (MMN), as a component of the CAEP, is an event-related potential that measures the difference between responses to rarely presented deviant stimuli and standard stimuli in a series. The expected latency is around 160–200 ms after the stimulus onset for acoustic hearing and differences over threshold. The occurrence of an MMN was explained as a mismatch between the deviant and the standard. It is assumed that the more frequent presentation of the standard forms a memory trace in the subject's brain, and the neural response becomes smaller due to repetition suppression^12^. Rare deviant tones reactivate adapted neurons which then react with a larger response^13^. The brain thus encodes regularities by encountering repeated stimuli and forming a sensory prediction about the incoming stimulus. The MMN following a new stimulus is interpreted as an error response to rare deviants which violate the prediction^14^. The sources of MMN are located in two different brain regions, the frontal and temporal cortices^15^. To elicit the MMN and avoid N2b contamination which reflects recruited attention, participants are asked to focus on another task like watching a silent movie^16^.

In normal hearing subjects, pitch, loudness, duration, timbre, and directional cues are known to elicit an MMN response^17^. An MMN response can also be measured in CI users and used to assess auditory perception^18–20^. MMN responses reflect preattentive discrimination, regardless of the subjects’ verbal or behavioral interaction. Therefore, measurement of MMN could be used to assess auditory perception in CI users who lack the ability to provide sufficient subjective feedback and thus allow for better fitting of the audio processor^18,21–23^.

In CI users, sound signals can be transmitted to the audio processor by free-field stimulation. In addition,direct electrical transmission of the audio processor is possible. This reduces interferences from acoustic preprocessing or influences of microphone and audio processor characteristics^19,22,24,25^. Direct electrical stimulation allows specific access to the implanted ear without the need to block or mask the better-hearing contralateral ear, as for example, in patients with unilateral deafness. Ponton and Don^22^ stimulated pairs of CI electrodes at the apical and basal ends of the electrode array in pediatric and adult CI users and elicited electrically evoked MMN (eMMN) responses by changing the length of a stimulus pulse train and pitch at the group level. For apical stimulation, the MMN latency was shorter compared to basal stimulation. Wable et al.^25^ compared eMMN responses elicited by stimulating different electrodes across the array starting at the apical end of the CI electrode array. No correlation was found between electrode spacing, i.e., the electrode distance between standard and deviant stimulation, and eMMN amplitudes or latencies.

In normal-hearing subjects, both loudness increases and decreases are appropriate cues to elicit a MMN, with MMN amplitude being greater for loudness increases^26–28^. McKay et al.^29^ psychophysically examined the intensity discrimination for individual electrodes in CI users and showed that the resolution of intensity differences is highest for medial electrodes. Scheperle et al.^30,31^ also used direct stimulation of Nucleus cochlear implants and investigated the spatial and spectral selectivity with acoustic change complexes in maps designed for the study. Their focus was on connections between peripheral and cortical potentials. They found evidence for spatial difference detection for neighboring electrode contacts in the cortical potentials. To the best of our knowledge, there is no data on eMMN with MED-EL devices regarding loudness deviations in CI users, either using direct stimulation or stimulation with an audio processor. Since the signal transmitted by the coil and the actual dimensions of parameters differ between the manufacturers, different artefacts have been observed^11^ and could influence the MMN detection. There are different strategies of signal transmission varying between continuous signal transmissions below threshold to transmissions only when signals occur. That leads to different artifacts. Beside these facts there is another difference one could investigate further with MED-EL devices; MED-EL uses longer CI electrode carriers compared to other manufacturers. Thus, more apical regions will be stimulated which could also provide new information about the discrimination ability in CI users for low-frequency tones.

Although MMN recording is not widely used in routine clinical practice in natural hearing individuals, it could be beneficial for CI users. In particular, it can be used to measure the progress in speech perception during auditory verbal therapy in noncooperative patients. Among other factors, speech perception depends on the ability to detect loudness differences and spectral features. Therefore, measurement of an MMN for loudness and spectral cues would contribute to the audiologic diagnostic process.

Herein, we focus on the recording of the eMMN, where the acoustic stimulus is sent directly into the audio processor. This method does require a dedicated cable for each audio processor or a wireless options, which can be cumbersome. However, delivering the stimulus in this manner is more controlled than via natural hearing through the processor and avoids the device processing of the auditory stimuli. Using a clinical setup for eMMN recording could be advantageous for implementing this measurement into clinical routine. As shown by Kranick et al.^32^, it is possible to record CAEPs with a clinical amplifier system.

The primary objective of this study was to electrically elicit an MMN to pitch and loudness cues generated by changes in CI electrode configuration and stimulation amplitude in MED-EL CI users. We decided to limit ourselves to this specific system in this work because it allows us to build on previous work^32^ and limit the influence of the experimental setup. The secondary objective was to demonstrate the applicability of recording eMMN in MED-EL CI users with a routine clinical recording setup.

## Materials and methods

### Participants

Participants were experienced adult CI users (≥ 6 months of CI use) with a Concerto or Synchrony CI device (MED-EL, Innsbruck, Austria). Exclusion criteria were having more than two deactivated CI electrodes, co-stimulation of the facial nerve, and alcohol or drug abuse, pregnancy, dementia, or concurrent participation in a pharmacological study. Participants were patients of the Halle Hearing and Implant Center and were recruited by letters, e-mails, or during routine clinical visits. All participants gave written informed consent. Protocols and consent forms were approved by the Ethics Committee of the Martin Luther University Halle–Wittenberg (Approval No.: 2020-124). All measurements were performed in accordance with all relevant guidelines. Participants received financial compensation for participation in the study with sessions lasting about 2 h.

### Stimuli

Before presentation of the stimuli, a study specific experimental fitting map was created for each participant to determine the most comfortable intensity (MCL) and threshold (THR) levels at electrodes 3, 4, 6, 9, and 10 using MAESTRO 8 clinical software (MED-EL, Innsbruck, Austria) and converted for use in Psyworks 5 software (MED-EL, Innsbruck, Austria). To avoid loudness cues between the selected electrodes, MCLs were balanced between these electrodes^33^.

For stimulation, electric bursts consisting of biphasic electrical pulses with an interstimulus duration of 1 ms and with a length of 75 ms, as used in everyday stimulation, were generated. Bursts were presented in a mixed oddball paradigm generated by the Psyworks 5 software as trains with a stimulus onset asynchrony of 350 ms (including a 5% jitter).

For the pitch cue, the stimulating electrode was switched between basal electrodes 9 (standard) and 10 (deviant) and apical electrodes 3 (standard) and 4 (deviant). Loudness was set to 80% of the dynamic range (MCL minus THR). For the loudness cue, the stimulation levels MCL and MCL minus 50% dynamic range were used as standard and deviant stimuli and delivered from electrode 6.

Pseudorandomized sequences for the eMMN elicitation were generated by a Python script (Python Software Foundation, version 3.7.6.). For each loudness and pitch cue condition, a block of 2000 stimuli was presented, containing 250 randomly deviant stimuli. Each block began with 12 standard stimuli, and each deviant stimulus was followed by at least 3 standard stimuli. Additional blocks were formed in which the roles of standard and deviant stimuli were switched. Presentation of each block took 15 min. A total of 6 blocks were presented in pseudorandomized order, and the measurement took approximately 1.5 h in total.

The stimulation parameters of the blocks are summarized in Table 1. Stimulation was performed using the RIB2DLL interface (University of Innsbruck, Austria). The MAX Programming Interface, which converts stimulation data into a CI data protocol, stimulated the CI via the MAX Coil.

**Table 1:** Summary of the stimulation conditions.

Stimulation condition	Standard	Deviant
Pitch cue—apical		
Deviant downward	Electrode 4	Electrode 3
Deviant upward	Electrode 3	Electrode 4
Pitch cue—basal		
Deviant upward	Electrode 9	Electrode 10
Deviant downward	Electrode 10	Electrode 9
Loudness cue		
Deviant downward	MCL	MCL minus 50% DR
Deviant upward	MCL minus 50% DR	MCL

*MCL* Most comfortable level; *DR* Dynamic range.

Because most clinical AEP recording systems have two memory buffers for averaging, the trigger signal was set to the last standard stimuli before any deviant. Thus, any system capable of recording clinical evoked potentials with alternating polarity would be able to automatically calculate the eMMN by storing the sweeps alternately in two buffers (A: standard and B: deviant) and subtracting them (B minus A). As a result, the averages in the buffers have comparable signal-to-noise-ratios (SNR)^32^.

### Data recording

**Figure 1:**
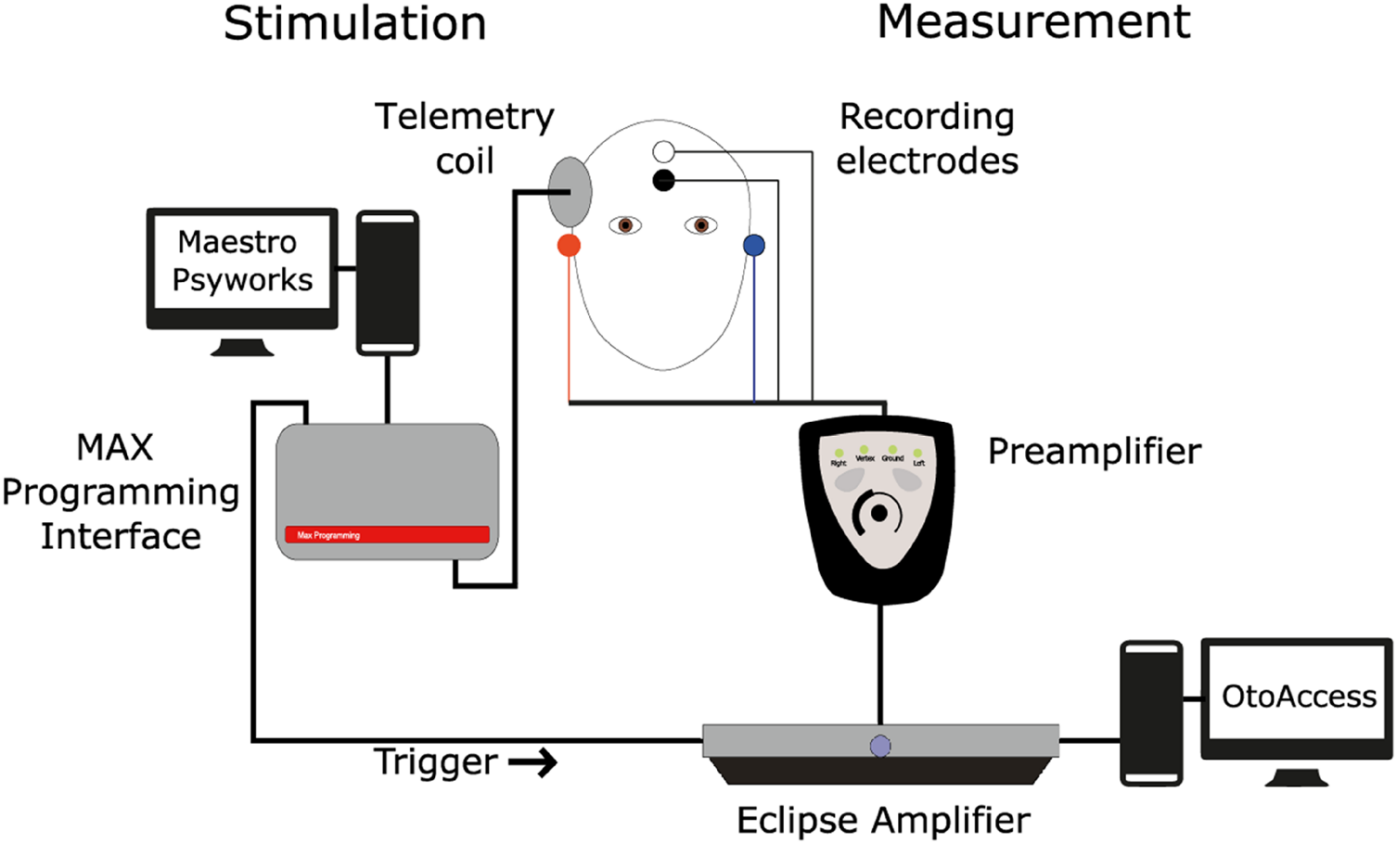
Setup for the EEG recording and stimulation.

The eMMN measurements were conducted in an acoustically and electrically shielded room. EEG was recorded using the 2-channels Eclipse EP25 amplification system (Interacoustics A/S, Middelfart, Denmark) at a sampling rate of 485 Hz. The trigger signal was generated by the MAX interface as described previously. Before attaching the recording electrodes (Ambu GmbH, Bad Nauheim, Germany), the skin was prepared with Nuprep (Weaver and Company, Aurora, USA), an abrasive gel. The active electrode was positioned on the vertex and referenced to the left and right mastoid, short-circuited using a jumper cable, with the ground electrode on the low forehead. The electrical connection of the left and right mastoid produced a neutral reference for the measurement of potential differences^34^. The impedances of the EEG electrode were kept below 3 kOhm. During the measurements, participants sat in a comfortable armchair, watched a silent movie with subtitles, and were instructed not to pay attention to the stimuli and to avoid movements. A recording protocol was used to store the EEG epochs following the standard and deviant stimuli in two different buffers. The data were filtered online with a high-pass filter of 1 Hz and a low-pass filter of 100 Hz (6 dB/octave), and no artifact rejection was used. See Fig. 1f or the EEG recording and stimulation setup.

### Data analysis

The recorded epochs stored in the standard and deviant buffers were exported from the Interacoustics software, and further data analysis was performed offline using custom Python scripts (Python Software Foundation, version 3.7.6.). Residual noise was calculated as root mean square (RMS) for each epoch to apply an artifact rejection limit of 40 µV. Epochs were then filtered using a Butterworth low-pass filter with zero phase shift 20 Hz (12 dB/octave)^34^.

**Figure 2:**
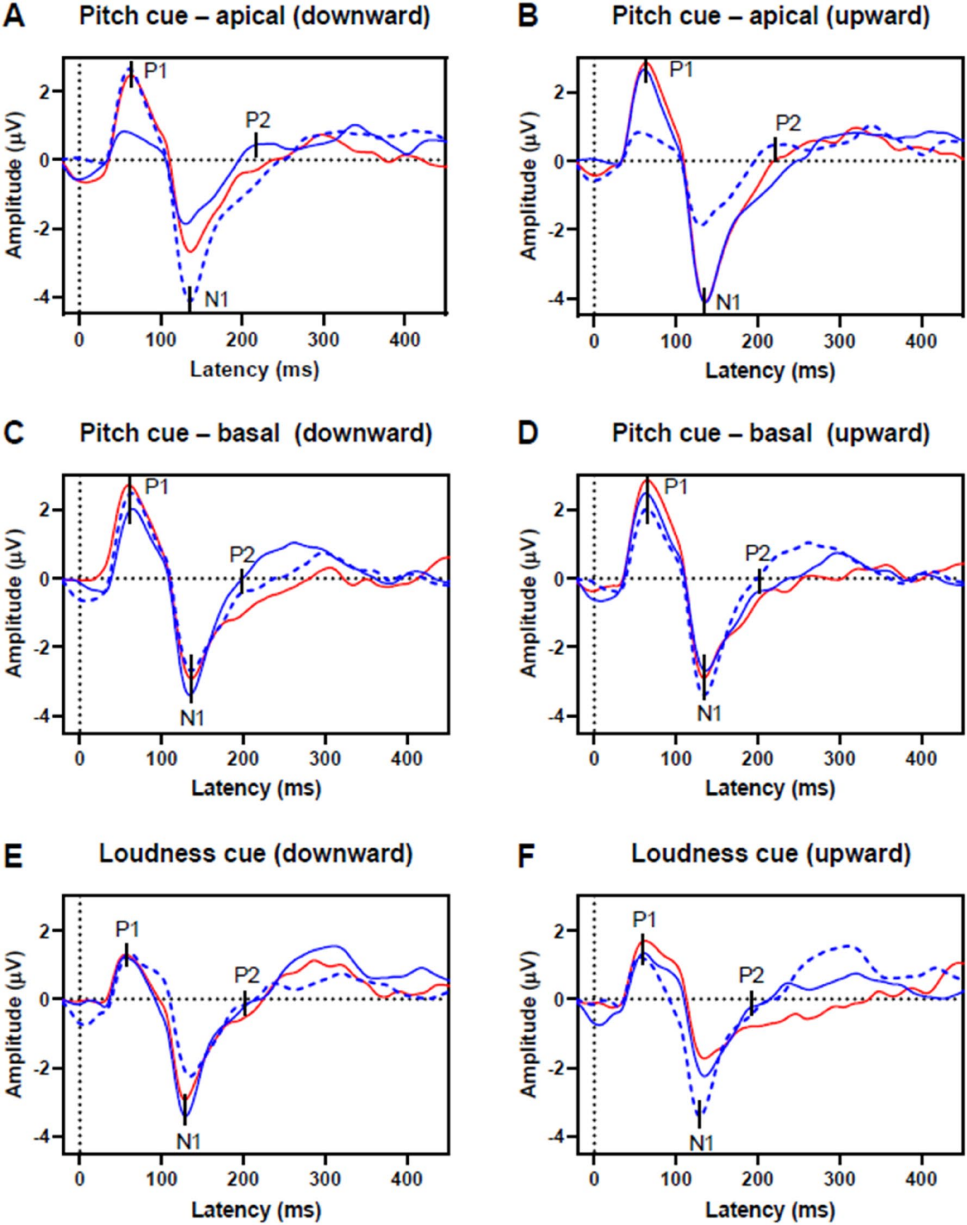
EEG responses for standard (blue) and deviant (red) stimulation as averages over all participants (n = 17). The deviant stimuli differed from the standard stimuli in pitch at an apical CI electrode (**A**, **B**), in pitch at a basal electrode (**C**, **D**) or loudness (**E**, **F**). For comparison the interblock standard response waveform (blue dashed line) is depicted that follows the physical identical standard stimuli to the deviant stimuli.

The eMMN waveforms were obtained by subtracting the response to the stimulus presented as standard from the response to the stimulus presented as deviant (deviant minus standard). EMMN responses can be measured as differences between deviant and standard responses of the same oddball paradigm (intrablock difference) or using the standard of an oddball block with reversed stimulus roles (interblock difference). For each stimulation condition and each eMMN calculation method (interblock and intrablock difference), the grand average waveforms were calculated as the mean of the respective individual responses to standard and deviant stimuli (see Fig. 2) Further analysis was done with the interblock differences.

The grand average eMMN responses by upward and downward deviants were combined to measure eMMN latencies as the most negative peak between latencies of 100 and 300 ms when an eMMN response was elicited. Individual standard and deviant amplitudes were then calculated as mean amplitudes within a time range of ± 20 ms centered around the grand average peak latency.

The distributions of standard and deviant amplitudes were descriptively reported. As a secondary analysis a comparison with a 3-way analysis of variance (ANOVA) for repeated measured with the within-subject factors condition (pitch cue apical, pitch cue basal, loudness cue), stimulus type (standard, deviant), and deviant direction (upward, downward) was done. Averaged standard and deviant responses were tested for normal distribution using the Kolmogorov–Smirnov test and for sphericity using the Mauchly test. Post hoc comparisons were performed with paired-sample *t*-tests. All statistical analyses were performed with SPSS software (Version 28, IBM, Armonk, NY, USA). Alpha was set to 5% and Bonferroni corrected.

The data sets analyzed in the current study are available from the corresponding author on request.

### Results

Twenty-one CI users (9 male, 12 female) were included in the study from January to May 2020. Participant demographics are shown in Table 2. After completion of the EEG recordings, four participants were excluded from the analysis because of large motion artifacts or because of partially detached recording electrodes. Thus, valid eMMN recordings from 17 participants were available for analysis.

**Table 2:** Demographic data of the participants.

Participant ID	Age [years]	Sex	Duration of non-serviceable hearing§ [years]	Aetiology	CI type and electrode	Implanted side	Deactivated electrodes	CI experience [years]
101*	69	M	3	Chronic otitis media	Mil 000 FLEX28	L		8
102	66	I.I	0	Progredient sensorineural hearing loss	Mil 250 FLEX28	L		1.5
103	80	F	0	Progredient sensorineural hearing loss	Mil 000 FLEX28	R	1, 12	4.5
104	68	M	unknown	Progredient sensorineural hearing loss	Mil 250 FLEX24	R	11, 12	0.5
105	84	F	0	Progredient sensorineural hearing loss and ISSHL	MM000 Standard	L		10
106	74	F	49	Measles infection	Mil 200 FLEX28	R	12	4.5
107*	42	F	1	Gentamicine intoxication	Mil 000 FLEX28	R		6.5
108	44	F	1	Susac syndrome	Mil 200 FLEX28	R	12	3.5
109	66	F	2	Progredient sensorineural hearing loss and ISSHL	MM000 Standard	R		9.5
110	50	F	37	Mumps infection	Mil000 FLEX28	L	11, 12	8.5
111	66	M	2	Chronic otitis media and fibrosis	Mil 200 FLEX28	L		6
112*	67	F	23	ISSHL	Mil 000 FLEX28	R		5.5
113	70	M	6	Progredient sensorineural hearing loss	Mil 000 FLEX28	L		8
114	69	F	13	Chronic otitis media in childhood	Mil 200 FLEX28	R		5.5
115	54	M	1	Chronic otitis media	Mil 000 FLEX28	L		8
116	69	M	1	ISSHL	Mil 000 FLEX28	R		7.5
117	77	F	2	Progredient sensorineural and conductive hearing loss	Mil 200 FLEX28	R		2
118	59	F	1	Meniere disease and progredient sensorineural hearing loss	Mil 250 FLEX28	L		1
119	77	M	8	Postinflammatory medial meatal fibrosis	Mil 200 FLEX28	R		5
120*	58	F	6	Large endolymph aqueduct syndrome	MM000 FORM24	R		6.5
121	77	M	10	Chronic otitis media	Mil000 FLEX28	R		7.5

*L* Left. *R* Right. *F* Female. *M* Male. *ISSHL* Idiopathic sudden sensorineural hearing loss.

*Removed from analysis because of detachached recording electrodes or technical issues. §within the audiological indication for a CI. which usually means a word recognition score of 50% or less.

Figure 2 shows the averaged response amplitudes for the standard and deviant stimuli. Distinct P1 and N1 CAEP components were elicited. No artifacts directly attributable to electrical stimulation were evident in the data.

**Figure 3:**
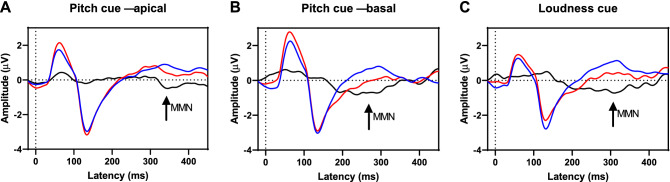
EEG responses for standard (blue) and deviant (red) stimulation as averages over all participants (n = 17) and over the deviance directions upward and downward. The black curve shows the deviant minus standard difference. The deviant stimuli differed from the standard stimuli in pitch at an apical CI electrode (**A**), in pitch at a basal electrode (**B**) or loudness (**C**). For pitch cues at a basal electrode and loudness cues significant differences were found and interpreted as eMMN response (arrows).

Table 3 shows the averaged amplitudes of the waveforms following standard and deviant responses in the latency range of the expected MMN. The Kolmogorov–Smirnov test confirmed a normal distribution of all individual distributions of standard and deviant responses. An ANOVA of these responses revealed significant main effects of the stimulus type (*F*(1,16) = 7.7, *p* = 0.014, η^2^ = 0.325) and the condition (*F*(2,15) = 4.74, *p* = 0.025, η^2^ = 0.387). Post-hoc comparisons showed lower amplitudes after deviant stimuli compared to standard stimuli. No other main effects or interactions were significant. Because the effect of deviance direction was not significant, both directions were combined for further analysis. The combined waveforms of standard, deviant, and difference are shown in Fig. 3. The negativity in the deviant minus standard difference waveforms for the *Pitch cue*—*basal* and *Loudness cue* conditions was interpreted as eMMN with measured latencies of 238 ms and 310 ms, respectively. Around these latencies, significant differences were found between standard and deviant stimuli for the *Pitch cue*—*basal* (− 0.75 µV (SD: 1.11), *t*(16) = 2.7, *p* = 0.014, *d* = 0.67) and *Loudness cue* (− 0.66 µV (SD: 1.18), *t*(16) = 2.3, *p* = 0.035, *d* = 0.56) conditions, but not for the *Pitch cue*—*apical* condition (*p* > 0.05, *d* =  − 0.14). Table 4 shows the standard, deviant and eMMN amplitudes.

**Table 3:** Mean amplitudes of the waveforms following standard and deviant responses in the latency range of the expected MMN.

Stimulation condition	Standard		Deviant	
	Mean (μV)	SD (μV)	Mean (μV)	SD (μV)
Pitch cue—apical				
Deviant downward	0.364	1.582	0.589	1.639
Deviant upward	0.464	1.415	0.561	1.508
Pitch cue—basal				
Deviant upward	− 0.045	1.274	− 0.259	1.229
Deviant downward	0.793	1.801	− 0.486	1.856
Loudness cue				
Deviant downward	1.483	1.237	0.989	1.733
Deviant upward	0.645	0.887	− 0.179	1.344

**Table 4:** Mean amplitudes following standard and deviant stimuli and difference amplitudes.

Stimulation condition	Standard		Devant		Difference
	Mean (μV)	SD (mV)	Mean (μV)	SD (μV)	Mean (μV)
Pitch cue—apical	0.414	1.281	0.575	1.283	0.161
Pitch cue—basal	0.374	1.223	− 0.372	0.961	− 0.746*
Loudness cue	1.064	0.794	0.405	1.013	− 0.660*

*p < 0.05.

## Discussion

In this study, a significant eMMN for pitch cues was found for basal electrode pairs. Negative deflection in the difference waveform was not significant when the pairs of apical electrodes were stimulated. All patients had complete insertion of the CI electrode array. However, the angle of insertion may vary due to the individual cochlear length and different electrode types. The pitch cue was elicited by stimulating adjacent electrodes in the cochlea. The evoked eMMN response suggests that in the basal cochlear regions, the pitch of the electrodes was preattentively discriminated. In adjacent apical electrodes, pitch percepts were likely equal between adjacent electrodes. Therefore, we could confirm the observation of Ponton et al.^35^, who found an eMMN response when the stimulated electrodes were changed. However, they used a Nucleus (Cochlear Ltd, Sydney, Australia) device and a much larger electrode distances (basal–apical) to elicit a pitch cue. The observed latencies by Ponton of 89 ms for the apical electrode pair and 114 ms for the basal electrode pair were significantly earlier than in the present study. The present study shows that eMMN can be elicited even at much smaller electrode distances and thus smaller pitch differences. Since the apical electrodes for MED-EL were of greater insertion depth compared to Nucleus devices the very apical region becomes overall less tonotopically which would have reduced MMN amplitude in our results.

In addition to the physical electrode spacing, other factors such as the insertion angle and the distance of the electrode array from the modiolus also have an influence on pitch perception. Because we presented adjacent electrodes to the participants, in contrast to Ponton et al.^35^, the crosstalk between the electrodes studied was probably greater in our setup. This may have additionally led to the lack of significance of our apical difference waveform and increased the discrimination effort, which would shift the eMMN in the basal region or completely cancel the eMMN at apical electrode pairs.

Wable et al.^25^ electrically evoked MMN in CI users by stimulating electrode pairs at three different physical electrode separations. Apical electrode 13 (from the Digisonic DX 10, a 15-electrode implant) was used in oddball paradigms alternating with a more basally located electrode (electrode 12, 10, or 8) for stimulation. In contrast to our results, significant eMMN response were elicited for all electrode separations. The average eMMN amplitude across all electrode separations and participants was − 2.8 μV with a latency of 140 ms^25^. Compared with the measured eMMN responses in our study, the negative peak was larger. The stimuli presented by Wable et al.^25^ may have been easier to discriminate than the pitch cue of our study because eMMN amplitude increases with variation in physical parameters, in this case spacing of electrodes used for stimulation. However, because the authors evaluated all electrode separations together, no information on eMMN significance could be reported separately for the adjacent electrode pair. Nevertheless, the larger spacing likely contributed to a higher significance than was achieved with our setup.

A distinct eMMN response was elicited to loudness cues. This indicates that, on average, participants were able to discriminate the difference in stimulation amplitude at electrode 6. The reported results are the first data to show an eMMN response in CI users to loudness cues. As with other auditory evoked potentials, intensity changes affect the morphology of the response waveform. Gates et al.^28^ demonstrated an MMN elicited to loudness cues in normal hearing subjects and found that the morphology of the MMN depended on the magnitude of the loudness deviation. Because only one intensity pair was used in our oddball paradigm, no conclusion can be drawn about this dependence based on the results of this study. However, it is reasonable to assume that the loudness growth function would also be represented by the MMN or eMMN amplitudes in CI users. If confirmed by future studies, this could be a valuable tool for programming CI systems. In contrast, eSRT measurement by triggering the reflex only measures the correlation to a single loudness value.

In the present study, no effect of deviation direction was measured. Gates et al.^28^ elicited MMN mainly to louder deviants. Tervaniemi et al.^27^ presented only intensity decreases as deviants to normal-hearing subjects and found larger MMN amplitudes at larger loudness decreases. In paradigms for measuring loudness cues, N1 amplitude may change with stimulation level and be misinterpreted as MMN in the difference waveform. Louder deviants would elicit increased N1 through an attentional process, as louder deviants are expected to involuntarily capture participants' attention^27^. In our study, no differences were found between the N1 amplitudes of standard and deviant responses.

Näätänen et al.^14^ were able to elicit MMN responses by pitch changes and by small loudness changes (+ 4 dB, − 6 dB) in normal-hearing participants. They found a significant MMN only for the loudness decrease, as the deviation was larger than for the increase. In a later study^36^, the MMN response was measured for equal magnitudes of loudness decreases and increases, suggesting that the elicitation of MMN is independent of the direction of loudness change.

The stimulation level, which was linearly scaled in units of current, was changed from MCL to MCL minus 50% DR. McKay et al.^29^ investigated the resolution of stimulation intensity in users of Nucleus CIs (Cochlear Limited, Sydney, Australia). They directly stimulated individual CI electrodes at different locations along the array and assessed the perception of intensity changes at each electrode based on behavioral measures. The measured threshold for intensity difference was smaller for the medial electrodes (9.5% of the DR) than for the basal (10.3% of the DR) or apical electrodes (12.1% of the DR). Compared with the results of the present study, the intensity change of 50% of the DR at the medial electrode was probably very easy for participants to detect.

MMN and eMMN responses can be measured as differences between deviant and standard responses of the same oddball paradigm (intrablock difference) or using the standard of an oddball block with reversed stimulus roles (interblock difference). That standard stimulus would be physical identical to the deviant stimulus. The use of the interblock difference requires an additional control condition that is time consuming and therefore could potentially limit clinical applicability. However, Peter et al.^37^ reported that the two methods yielded similar MMN estimations for pitch cues. For tone duration cues, they observed a more significant MMN response with the interblock method. Calculating MMN using physically identical stimuli would avoid the influence of acoustic differences between standard and deviant stimuli, which may override the MMN response^25,37^. In the present study, only the interblock method was used to assess clinical applicability, where a shorter measurement time is required.

While no significant eMMN was elicited at apical electrodes in response to the pitch cue, it was elicited but at basal electrodes. The use of eMMN measurements to assess the ability of CI users to discriminate adjacent electrodes has limitations. An electrode spacing of one electrode (1.92 mm, 2.4 mm and 2.8 mm with MED-EL CI electrode arrays FLEX24, FLEX28, and STANDARD, respectively) may not be sufficient to elicit an eMMN, particularly in the apical region. Regarding loudness cues, the experimental design may be appropriated to measure participants’ loudness discrimination ability. However, additional loudness variances should be tested in eMMN experiments before further conclusions can be drawn. In addition, we have shown that eMMN responses can be recorded with a clinical amplification system, so this measurement may be easier to perform as part of routine clinical practice. When further knowledge about the correlation between eMMN morphology and intensity as well as pitch changes is gained in research setups, eliciting eMMN by other than oddball paradigms may be a next, promising step towards clinical applications.

Our results are consistent with a previous study reporting the ability to record cortical auditory evoked potentials with different clinical amplifiers in CI users and the small effect of stimulation artifact on the procedure^32^. In addition, studies that used multiple active recording electrodes obtained more detailed results because they were able to record EEG from multiple cortical sources simultaneously and apply specific artifact reduction algorithms to their data^24,38^. There are a few studies using clinical amplifiers for those more complex research questions^30,31^. Our recording setup with the clinical amplifier is one further promising setup because it demonstrates that detection of eMMN for loudness and pitch cues is not limited to research settings and can be performed with common clinical equipment, even if a specific stimulation-triggering paradigm must be used. Before eMMN measurements can be applied in a clinical context, a reliable, statistically objective method for eMMN detection at the single-subject level must be developed^39^.

The use of the eMMN could be used in the future for programming audio processors. To do this, the amplitude growth functions for the cues would first have to be determined as reference values. Recognizing loudness and frequency differences is essential for speech perception. Thus, information about the cortical processing of these differences is relevant for the assessment of the rehabilitation success. Based on this information, e.g. frequency allocation tables or stimulation levels could be adjusted.

## Limitations

Although we tried to keep the participants occupied during the study via silent movies, many of them got bored or accidently fell asleep. One participant was very restless and even stood up during the measurement, which made the recording unusable. Furthermore, some participants reported that the frequent repetition of the tones was very unpleasant, others found it soothing. Especially in the early time period of CI use, people tend to feel uncomfortable and overwhelmed by the new hearing impressions.

A large number of stimulation trials, accompanied by a long duration, is inevitable for a sufficient SNR. As this study was exploratory using six test patterns, using a different sequence for eMMN elicitation could improve the efficiency by reducing the overall recording time. Gates et al.^28^ used a “intensity dependency paradigm”, where one standard intensity is interspersed with three other intensities serving as deviants, in a single recording session to evoke MMN from three different stimuli within the same task in normal hearing listeners. Similarly, Näätänen et al.^40^ demonstrated that presenting more than one deviant per recording block to normal hearing listeners could feasibly decrease the recording time. Contrary to Gates et al.^28^, they varied five instead of one stimulation parameter per block.

## Conclusions

This study demonstrates that eMMN responses can be recorded in CI subjects using a measurement system from routine clinical practice. Loudness cues could be reliably used to elicit eMMN if the intensity deviance was 50% of the dynamic range. For pitch cues, an electrode spacing of one electrode was not sufficient to elicit an eMMN.
